# Comparison of Bone Mineral Density Between Veterans and Non-Veterans and Its Impact on Fracture Risk Assessment

**DOI:** 10.3390/medicina60111811

**Published:** 2024-11-04

**Authors:** Ya-Lien Deng, Chun-Sheng Hsu, Yi-Ming Chen, Shih-Yi Lin, Tse-Yu Chen, Chi-Ruei Li, Hsu-Tung Lee, Ying-Chia Wu

**Affiliations:** 1 Osteoporosis Prevention Center, Taichung Veterans General Hospital, Taichung 407219, Taiwan; cfalsm@vghtc.gov.tw; 2 Department of Nursing, Taichung Veterans General Hospital, Taichung 407219, Taiwan; 3 Department of Physical Medicine and Rehabilitation, Taichung Veterans General Hospital, Taichung 407219, Taiwan; chincent@vghtc.gov.tw; 4 Department of Post-Baccalaureate Medicine, College of Medicine, National Chung Hsing University, Taichung 402202, Taiwan; ymchen1@vghtc.gov.tw (Y.-M.C.); leesd2001@hotmail.com (H.-T.L.); 5 Department of Medical Research, Taichung Veterans General Hospital, Taichung 407219, Taiwan; 6 Division of Allergy, Immunology and Rheumatology, Taichung Veterans General Hospital, Taichung 407219, Taiwan; 7 Center for Geriatrics and Gerontology, Taichung Veterans General Hospital, Taichung 407219, Taiwan; sylin@vghtc.gov.tw; 8 Department of Neurosurgery, Neurological Institute, Taichung Veterans General Hospital, Taichung 407219, Taiwan; chentseyu@vghtc.gov.tw (T.-Y.C.); fantastic1694@gmail.com (C.-R.L.); 9 Doctoral Program in Translational Medicine, National Chung Hsing University, Taichung 402202, Taiwan; 10 Institute of Medicine, Chung Shan Medical University, Taichung 402306, Taiwan; 11 Division of Neurosurgical Oncology, Neurological Institute, Taichung Veterans General Hospital, Taichung 407219, Taiwan; 12 Lee’s Medical Corporation, Taichung 437, Taiwan

**Keywords:** bone mineral density, fracture, mortality, osteoporosis, veteran

## Abstract

*Background and Objectives*: Osteoporosis poses significant health risks, especially among veterans, due to lifestyle factors. This study compares bone density and fracture risks between male veterans and non-veterans. *Materials and Methods*: We conducted a retrospective analysis of 1427 veterans from the Taichung Veterans General Hospital osteoporosis database (2010–2022), matched 1:1 by age and gender with non-veterans. Bone mineral density (BMD) was measured via dual-energy X-ray absorptiometry (DXA). Comorbidities, fracture sites, and mortality data were tracked. Conditional logistic regression was used to identify factors influencing fracture risk. *Results*: The study found that veterans have a higher fracture risk (univariable OR 1.24, multivariable OR 1.20, *p* < 0.001). Fracture victims were slightly younger in the veterans group (78.7 ± 10.0 years vs. 80.1 ± 9.2 years, *p* = 0.010), who also had better T-scores in the lumbar spine and left femoral neck. Veterans showed a higher post-fracture mortality rate (39.9% vs. 31.9%, *p* = 0.001) and a greater incidence of radial fractures (5.01% vs. 2.96%, *p* = 0.036). Importantly, veterans exhibited a trend toward more hip fractures compared with non-veterans (27.0% vs. 23.6%, *p* = 1.017), suggesting a potential difference despite not reaching statistical significance. *Conclusions*: In the present study, we found that veterans have higher rates of comorbidities, and higher mortality after a fracture event, highlighting the need for targeted medical interventions to address these differences. Further intervention to prevent avoidable fractures and the provision of adequate care for long-term osteoporosis management remain critical issues.

## 1. Introduction

Osteoporosis is a common disease among the elderly. Therefore, prevention is a major public health issue worldwide. Previous studies have reported that the risk factors for osteoporosis include inadequate exercise, inadequate nutrition such as a lack of calcium and vitamin D, early menopause, smoking, and alcohol abuse [1]. In addition, lifestyle-related factors such as dairy [2], sleep pattern and duration [3,4] and physical activity [5] also have influence on bone quality.

Resulting from low bone mineral density (BMD), osteoporotic fractures lead to decreased quality of life, morbidity, disability, and mortality [1]. Huge socioeconomic burdens due to increased healthcare costs and care requirements are inevitable world-wide issues [6]. In the United States, the annual average healthcare resource utilization and costs were USD 19,470 for osteoporosis-related treatments and services in 2020 [7]. According to the ICUROS study [8], a recent prospective micro-costing study conducted in France, the total direct and indirect costs over eighteen months (including initial fracture-related costs and follow-up costs) were EUR 23,926 for hip fractures (EUR 15,951 per annum) and EUR 14,561 for vertebral fractures (EUR 9707 per annum). An analytical study based on the National Health Insurance Research Database in Taiwan in 2016 focusing on postmenopausal women demonstrated that patients experiencing osteoporotic fractures were at considerable excess risk of death and incurred substantially higher treatment costs, notably for hip fractures [9].

Among the elderly cohort, veterans generally have a higher risk of developing osteoporosis due to their lifestyles and health issues related to military service [10,11]. In the discourse on the biopsychosocial aspects of health and aging, several ways in which the social environment impacts health have been highlighted. These include individual social relationships, socioeconomic status, and community characteristics. Veterans may differ from non-veterans in many physical and sociocultural factors, which could increase the rate of fractures. Such factors include demographic characteristics, social challenges and stressors, and health behaviors [12,13]. Furthermore, due to the historical background of veterans in Taiwan, many of whom migrated from China for military purposes at a young age, there are additional impacts on their health. This migration has influenced their marital status and educational attainment, and has often resulted in inadequate familial support or them even living alone, all of which can significantly affect health care outcomes.

The aim of our study is to compare bone density differences between male veterans and non-veterans, and to explore the related factors influencing fracture incidence. This will help provide appropriate prevention and treatment strategies for veterans. Medical professionals can develop individualized prevention and treatment plans based on the veterans’ specific circumstances to reduce fracture risk and improve quality of life.

## 2. Materials and Methods

### 2.1. Enrolled Participants

We conducted a retrospective data analysis using the osteoporosis database of Taichung Veterans General Hospital in Taiwan. Our osteoporosis database included diagnoses of osteoporosis identified through the International Classification of Diseases, Ninth Revision, Clinical Modification (ICD-9-CM) and Tenth Revision (ICD-10), with diagnostic codes for osteoporosis (733.0, 733.00, 733.01, 733.02, 733.03, 733.09/M80–82) or osteoporotic fractures, including vertebral fractures (805.2–805.9/S22, S32), hip fractures (820.x/S72), and radial fractures (813.x/S52, S62). Patients with a history of traumatic incidents in their medical records were excluded. In this study, we retrospectively analyzed 1427 veterans who underwent their first dual-energy X-ray absorptiometry (DXA) scan at Taichung Veterans General Hospital between January 2010 and December 2022. Veterans were defined as individuals who had served on active duty for a period exceeding 10 years. Veteran patients were matched with non-veteran patients by age and gender in a 1:1 ratio. The exclusion criteria included female patients, and only bone density data within the two years before and after the first fracture were included in the analysis. We then retrospectively tracked demographic data, fracture sites, bone density, and chronic diseases from electronic health records. This study was approved by the Clinical Research Ethics Committee of Taichung Veterans General Hospital (CF18067B). As patient data were anonymized before analysis, written informed consent from patients was not required, and ethical approval was obtained.

### 2.2. Comorbidities and Mortality

We analyzed comorbidities such as osteoarthritis (M15–M19/715–716), rheumatoid arthritis (M05–M07, M08.001–M08.48/714), diabetes mellitus (E08–E13/250), hypertension (I10–I15/401–405), stroke (I60–I63, I69/430–434, 438), chronic kidney disease (N18/585), coronary artery disease (I20–I25/410, 412, 414), and peripheral artery disease (I70–I71/440, 443). The assessment of these comorbidities, along with the overall health status of the participants, was conducted to evaluate their potential impact on bone health and fracture risk. Lastly, we only took death after a fracture event into account for mortality evaluation.

### 2.3. Fracture Identification

For further analysis, data on the incidence and sites of osteoporotic fractures that occurred between January 2010 and December 2022 were extracted from a single institution. The osteoporotic fractures included vertebral fractures, hip fractures, and distal radial fractures, such as Colles fractures. Participants who sustained fractures due to traffic accidents or high-impact trauma (ICD-9-CM codes E810–E819, E881–E883, E884), pathological fractures (733.14, 733.15/M84), or those diagnosed with Paget’s disease (731.0/M88) were excluded from the analysis.

### 2.4. BMD Measurements

BMD measurements of the bilateral femoral necks and lumbar spine (L1–L4) were obtained using dual-energy X-ray absorptiometry (DXA) with the Lunar Prodigy system (General Electric, Fairfield, CT, USA), and results were expressed in g/cm^2^. BMD assessments were conducted prior to the initiation of anti-osteoporotic medications. The least significant change (LSC) was defined as ±0.010 g/cm^2^ for the lumbar spine (L1–L4) and ±0.012 g/cm^2^ for the femoral neck. T-scores were calculated based on the manufacturer’s reference data. In accordance with the World Health Organization (WHO) diagnostic criteria [14], osteoporosis was defined as a T-score ≤ −2.5; low bone mass as a T-score between −1.0 and −2.5; and normal bone density as a T-score > −1.0.

### 2.5. Statistical Analysis

Demographic data for continuous variables are presented as mean (standard deviation, SD), while categorical variables are expressed as the number (percentage) of patients. The Mann–Whitney U test was used to compare variables between veterans and the general population and to assess the association with bone density. Conditional logistic regression was employed to identify key factors influencing fracture risk. All analyses were conducted using the Statistical Package for the Social Sciences (SPSS), version 22.0 (IBM Corp., Armonk, NY, USA). A *p*-value of <0.05 was considered statistically significant.

## 3. Result

### 3.1. Demographic Data

In this study, we collected demographic variables, fracture sites, bone density values, clinical blood test results, and relevant chronic comorbidities from 1427 patients who discontinued osteoporosis medication. The basic statistical analysis results are organized as shown in Table 1.

From Table 1, we can observe the following findings. The table compares various health parameters between veterans and non-veterans, revealing several key findings. Firstly, there is no significant difference in age between the two groups (*p* = 1.000). However, veterans have lower lumbar spine bone mineral density (BMD) (*p* = 0.032), while there is no significant difference in T-scores and trabecular bone scores (TBS). In terms of laboratory data, veterans have higher phosphate levels (*p* = 0.002) and lower creatinine levels (*p* = 0.009). Additionally, comorbidities including osteoarthritis, rheumatoid arthritis, diabetes, hypertension, stroke, chronic kidney disease, coronary artery disease, and peripheral artery disease rates are significantly higher in the veterans group.

Veterans also have a higher rate of calcium acetate usage (*p* < 0.001) and a significantly higher mortality rate (*p* < 0.001). Regarding fracture sites, veterans exhibit a trend toward more hip fractures (27.0% vs. 23.6%), suggesting a potential difference despite not reaching statistical significance (*p* = 0.107). In addition, veterans have a higher incidence of radial fractures (*p* = 0.036).

### 3.2. Conditional Logistic Regression Analysis of Factors Associated with Fractures in Veterans

Table 2 presents a conditional logistic regression analysis identifying factors associated with fractures in veterans. Veterans show a higher fracture risk (univariable OR 1.24, multivariable OR 1.20, *p* < 0.001), while higher lumbar spine and femoral neck T-scores are protective (both *p* < 0.001). Hypertension increases fracture risk in the univariable model (OR 1.25, *p* = 0.002) but not in the multivariable model (OR 1.11, *p* = 0.296). Stroke (OR 1.22, *p* = 0.019) and PAD (OR 1.46, *p* = 0.018) are significant only in the univariable model. Calcium acetate (OR 1.44, *p* < 0.001) and alfacalcidol (OR 1.33, *p* = 0.009) increase the risk in the univariable model. The multivariable analysis confirms veteran status and lower lumbar spine T-scores as key risk factors.

### 3.3. Mann–Whitney U Test Results Comparing Veterans and Non-Veterans with Fractures

Table 3 presents the results of the Mann–Whitney U test, comparing various parameters between veteran and non-veteran fracture patients. Fracture victims are slightly younger in the veterans group than non-veterans (*p* = 0.010). In terms of BMD, fracture patients in the veterans group have higher BMD in the lumbar spine (*p* = 0.024), left femoral neck (*p* = 0.002), and right femoral neck (*p* < 0.001). Additionally, veterans have better T-scores in the lumbar spine (*p* < 0.001), left femoral neck (*p* < 0.001), and right femoral neck (*p* < 0.001). While there is no significant difference in the trabecular bone score (TBS) in terms of BMD (*p* = 0.079), veterans have better T-scores (*p* = 0.010). There is no significant difference in BMI between the two groups (*p* = 0.075). In laboratory data, veterans have higher phosphate levels (*p* = 0.026) and lower levels of creatinine (*p* = 0.005), albumin (*p* < 0.001), and alkaline phosphatase (ALP) (*p* = 0.012). Regarding fracture sites, veterans have a higher incidence of radial fractures (*p* = 0.036). A trend was noticed that veterans have a higher incidence of hip fractures compared with non-veterans (27.0% vs. 23.6%, respectively), although this difference did not reach statistical significance (*p* = 0.107). The incidence rates of osteoarthritis, rheumatoid arthritis, diabetes, hypertension, stroke, cataracts, chronic kidney disease, coronary artery disease, and peripheral artery disease are higher in the veterans group. In addition, veterans have higher usage rates of calcium and alfacalcidol. Lastly, higher mortality after a fracture event was noted in the veterans group (*p* = 0.001).

## 4. Discussion

In this study, we observed that veterans experience higher rates of comorbidities and increased mortality following fracture events compared with non-veterans, underscoring the urgent need for targeted medical interventions tailored to this population. Despite evidence of better bone health in the veteran fracture patients, they still exhibit a higher mortality rate, suggesting that factors beyond bone density, such as the burden of comorbidities and possibly delayed or inadequate post-fracture care, may contribute to this outcome. Notably, veterans are found to have a higher incidence of hip and radial fractures, while the incidence of spine fractures remains comparable to that of non-veterans.

Veteran fracture patients, despite potentially having better bone density, still exhibit a higher mortality rate. This paradox may be explained by the presence of comorbidities such as musculoskeletal disorders, acute or chronic cardiovascular diseases, stroke, and chronic kidney disease, which significantly influence mortality. These comorbidities are often the result of distinct lifestyle factors, dietary habits, and biopsychosocial influences that differentiate veterans from the non-veteran elderly population [10,11,15,16,17,18]. For instance, the high prevalence of smoking in veterans is a well-documented contributor to various health issues, including cardiovascular disease and respiratory conditions [19]. Similarly, obesity, driven by specific dietary and lifestyle patterns, is another critical risk factor that negatively impacts mobility and further elevates mortality risk within this group [11]. These findings emphasize the complex interplay of health behaviors and comorbidities in determining mortality outcomes in veterans with fractures.

Our study found that veterans have a higher incidence of hip fractures compared with non-veterans (27.0% vs. 23.6%, respectively), although this difference did not reach statistical significance (*p* = 0.107). Despite the lack of statistical significance, the observed trend aligns with existing literature, which suggests that hip fractures are particularly prevalent among elderly populations, including veterans. Hip fractures are not only more common in veterans but are also associated with a significantly higher mortality rate, especially among the elderly. The one-year mortality rate following a hip fracture has been reported to range between 14% and 58% [20,21,22,23], depending on factors such as age, overall health, and the presence of comorbid conditions. This high mortality rate is often attributed to the substantial loss of mobility that typically follows a hip fracture, leading to a loss of independence and an increased financial burden. These factors contribute to a 4% per year increase in the relative risk of mortality in the elderly population [24]. Focusing specifically on geriatric veterans, Bass et al. [25] reported those one out of three elderly male veterans who suffered a hip fracture died within one year, with the risk of mortality continuing to increase beyond six months after the initial injury. Additionally, a retrospective study by LaFleur et al. [17] suggested that female veterans had a higher incidence of hip fractures compared with non-veteran women with osteoporosis, despite the absence of well-established fracture risk factors.

These associated characteristics likely contribute to the increased risk of hip fractures in patients residing in nursing institutions. A 2020 report demonstrated that the adjusted proportion of residents who experienced at least one hospital transfer for a serious fracture between 2011 and 2016 in U.S. cohorts remained at approximately 2.5% [26]. According to data from the Taiwan Veterans Affairs Council, there are 5317 veterans currently living in veterans homes. While detailed data on hospital transfers due to fracture incidents in these veterans is lacking, the prevention of avoidable fractures and the provision of adequate care for long-term osteoporosis management remain critical issues. Addressing these concerns is crucial for improving quality of life and reducing the morbidity and mortality associated with fractures in this vulnerable population. For instance, a 2024 study found that antihypertensive medications were linked to an increased risk of fractures in older veterans residing in Veterans Health Administration nursing homes [26]. This highlights the importance of exercising caution and providing additional monitoring when initiating medical treatment for this at-risk group.

However, this study has several limitations. Its retrospective design makes the data vulnerable to entry errors and miscoding. Also, there may be biases related to the initial treatment decisions, as well as confounding factors due to indication. Additionally, important psychosocial factors such as economic status, family support, and physical activity were not included in the present analysis. The absence of these variables may limit the ability to fully understand the broader context of fracture risk and recovery in the study population. Furthermore, the comparison of BMD was conducted using the Mann–Whitney U-test, which does not allow for adjustments for variables such as BMI, age, and comorbidities.

## 5. Conclusions

In this study, we found that veterans have higher rates of comorbidities and increased mortality following a fracture, underscoring the need for targeted medical interventions to address these disparities. Given the poor outcomes associated with fractures in this population, it is essential to prioritize these concerns to improve quality of life and reduce the morbidity and mortality rates in this vulnerable group.

## Figures and Tables

**Table 1:** Comparison of veterans and non-veterans.

	Non-Veterans (n = 1427)		Veterans (n = 1427)		*p* Value
	Mean	±SD	Mean	±SD	
Age	76.58	±10.92	76.58	±10.92	1.000
Bone mineral density(g/cm^2^)					
Lumbar spine	1.098	±0.225	1.077	±0.218	0.032 *
Left femoral neck	0.766	±0.151	0.759	±0.147	0.121
Right femoral neck	0.765	±0.151	0.758	±0.146	0.156
T-score					
Lumbar spine	−0.335	±1.857	−0.326	±1.816	0.815
Left femoral neck	−1.841	±1.095	−1.812	±1.084	0.635
Right femoral neck	−1.851	±1.116	−1.818	±1.075	0.539
Trabecular bone score					
Bone mineral density	1.336	±0.099	1.344	±0.116	0.193
T-score	−0.963	±0.844	−0.845	±0.876	0.056
Body mass index (kg/m^2^)	23.91	±3.81	24.31	±3.73	0.083
Laboratory data					
25-OH-Vitamin D	28.65	±10.03	24.70	±9.30	0.179
Calcium	8.90	±0.70	8.84	±0.66	0.085
Phosphate	3.61	±0.87	3.43	±0.81	0.002 **
Creatinine	1.57	±1.92	1.28	±1.04	0.009 **
Intact PTH	139.24	±281.22	102.10	±143.14	0.764
Albumin	18.80	±243.51	8.82	±116.17	0.624
Alkaline phosphatase	136.04	±244.25	120.61	±117.54	0.093
Comorbidity					
Osteoarthritis	167	(11.70%)	420	(29.43%)	<0.001 **
Rheumatoid arthritis	37	(2.59%)	34	(2.38%)	0.718
Diabetes mellitus	262	(18.36%)	348	(24.39%)	<0.001 **
Hypertension	401	(28.10%)	690	(48.35%)	<0.001 **
Stroke	150	(10.51%)	264	(18.50%)	<0.001 **
Cataract	46	(3.22%)	263	(18.43%)	<0.001 **
Chronic kidney disease	236	(16.54%)	290	(20.32%)	0.009 **
Coronary artery disease	220	(15.42%)	416	(29.15%)	<0.001 **
Peripheral artery disease	42	(2.94%)	77	(5.40%)	0.001 **
Congestive heart failure	94	(6.59%)	155	(10.86%)	<0.001 **
Cirrhosis	15	(1.05%)	14	(0.98%)	0.852
Calcium supplement					
Carbonate	51	(3.57%)	105	(7.36%)	<0.001 **
Acetate	277	(19.41%)	398	(27.89%)	<0.001 **
Phosphate	226	(15.84%)	219	(15.35%)	0.718
Vitamin D supplement	125	(8.76%)	159	(11.14%)	0.033 *
Death	333	(23.34%)	533	(37.35%)	<0.001 **
Fracture sites	743	(52.07%)	918	(64.33%)	<0.001 **
Hip	175	(23.55%)	248	(27.02%)	0.107
Spine	507	(68.24%)	624	(67.97%)	0.909
Radius	22	(2.96%)	46	(5.01%)	0.036 *

Mann–Whitney U test. Chi-Square test. * *p* < 0.5, ** *p* < 0.01.

**Table 2:** Conditional regression of independent factors associated with fracture of veterans.

	Simple Model			Multiple Model		
	OR	(95% CI)	*p* Value	OR	(95% CI)	*p* Value
Veterans	1.24	(1.12–1.36)	<0.001 **	1.20	(1.06–1.36)	0.005 **
Age	-					
Bone mineral density(g/cm^2^)						
Lumbar spine	0.42	(0.29–0.61)	<0.001 **	0.39	(0.27–0.58)	<0.001 **
Left femoral neck	0.16	(0.09–0.29)	<0.001 **			
Right femoral neck	0.18	(0.10–0.32)	<0.001 **			
T-score						
Lumbar spine	0.91	(0.87–0.95)	<0.001 **			
Left femoral neck	0.79	(0.73–0.85)	<0.001 **			
Right femoral neck	0.80	(0.75–0.87)	<0.001 **			
Comorbidity						
Osteoarthritis	1.18	(1.01–1.38)	0.042 *	1.03	(0.84–1.28)	0.748
Rheumatoid arthritis	1.08	(0.70–1.67)	0.739			
Diabetes mellitus	1.11	(0.94–1.30)	0.205			
Hypertension	1.25	(1.09–1.43)	0.002 **	1.11	(0.91–1.35)	0.296
Stroke	1.24	(1.04–1.48)	0.019 *	1.17	(0.93–1.47)	0.172
Cataract	1.18	(0.97–1.44)	0.106			
Chronic kidney disease	1.20	(1.01–1.42)	0.035 *	1.11	(0.90–1.38)	0.339
Coronary artery disease	1.20	(1.03–1.40)	0.020 *	1.06	(0.87–1.30)	0.548
Peripheral artery disease	1.46	(1.07–2.00)	0.018 *	1.28	(0.87–1.88)	0.206
Congestive heart failure	1.19	(0.95–1.49)	0.124			
Cirrhosis	1.50	(0.80–2.82)	0.209			
Calcium supplement						
Carbonate	1.08	(0.81–1.45)	0.601			
Acetate	1.44	(1.23–1.67)	<0.001 **			
Phosphate	1.54	(1.29–1.84)	<0.001 **			
Vitamin D supplement	1.33	(1.07–1.65)	0.009 **			

Conditional logistic regression. * *p* < 0.05, ** *p* < 0.01.

**Table 3:** Comparison of fracture groups between veterans and non-veterans.

	Non-Veterans (n = 743)		Veterans (n = 918)		*p* Value
	Mean	±SD	Mean	±SD	
Age	80.05	±9.22	78.66	±10.00	0.010 *
Bone mineral density (g/cm^2^)					
Lumbar spine	1.023	±0.212	1.048	±0.220	0.024 *
Left femoral neck	0.704	±0.144	0.728	±0.141	0.002 **
Right femoral neck	0.703	±0.142	0.729	±0.139	<0.001 **
T-score					
Lumbar spine	−0.96	±1.77	−0.56	±1.83	<0.001 **
Left femoral neck	−2.29	±1.04	−2.04	±1.04	<0.001 **
Right femoral neck	−2.30	±1.04	−2.02	±1.06	<0.001 **
Trabecular bone score					
Bone mineral density	1.314	±0.099	1.326	±0.128	0.079
T-score	−1.183	±0.861	−0.979	±0.905	0.010 *
Body mass index (kg/m^2^)	23.44	±4.11	24.00	±3.76	0.062
Laboratory data					
25-OH-Vitamin D (mg/dL)	27.73	±11.20	22.76	±6.31	0.309
Calcium (mg/dL)	8.79	±0.75	8.71	±0.65	0.242
Phosphate (mg/dL)	3.50	±0.85	3.39	±0.79	0.295
Creatinine(mg/dL)	1.53	±1.63	1.26	±0.93	0.006 **
Intact PTH (mg/dL)	116.34	±245.74	93.81	±109.57	0.610
Albumin (mg/dL)	18.95	±207.09	11.58	±146.40	0.012 *
Alkaline phosphatase (mg/dL)	136.80	±96.23	124.05	±130.54	<0.001 **
Comorbidity					
Osteoarthritis	107	(14.40%)	317	(34.53%)	<0.001 **
Rheumatoid arthritis	17	(2.29%)	25	(2.72%)	0.574
Diabetes mellitus	147	(19.78%)	245	(26.69%)	0.001 **
Hypertension	245	(32.97%)	493	(53.70%)	<0.001 **
Stroke	113	(15.21%)	203	(22.11%)	<0.001 **
Cataract	36	(4.85%)	195	(21.24%)	<0.001 **
Chronic kidney disease	155	(20.86%)	218	(23.75%)	0.161
Coronary artery disease	130	(17.50%)	307	(33.44%)	<0.001 **
Peripheral artery disease	31	(4.17%)	67	(7.30%)	0.007 **
Congestive heart failure	71	(9.56%)	115	(12.53%)	0.056
Cirrhosis	12	(1.62%)	12	(1.31%)	0.601
Calcium supplement					
Carbonate	29	(3.90%)	72	(7.84%)	0.001 **
Acetate	203	(27.32%)	320	(34.86%)	0.001 **
Phosphate	191	(25.71%)	192	(20.92%)	0.021 *
Vitamin D supplement	86	(11.57%)	130	(14.16%)	0.119
Death	237	(31.90%)	366	(39.87%)	0.001 **
Fracture sites					
Hip	175	(23.55%)	248	(27.02%)	0.107
Spine	507	(68.24%)	624	(67.97%)	0.909
Radius	22	(2.96%)	46	(5.01%)	0.036 *

Mann–Whitney U test. Chi-Square test. * *p* < 0.5, ** *p* < 0.01.

## Data Availability

Data are available upon reasonable request. The datasets used during the current study are available from the Taichung Veterans General Hospital; however, restrictions apply regarding the availability of these data, as they are not publicly available. However, the data are available from the corresponding author upon reasonable request and with permission from the Taichung Veterans General Hospital.
